# Social media use among adolescents with eating disorders: a double-edged sword

**DOI:** 10.3389/fpsyt.2024.1300182

**Published:** 2024-02-09

**Authors:** Faisal A. Nawaz, Mehr Muhammad Adeel Riaz, Nimrat ul ain Banday, Aakanksha Singh, Zara Arshad, Hanan Derby, Meshal A. Sultan

**Affiliations:** ^1^ Al Amal Psychiatric Hospital, Emirates Health Services, Dubai, United Arab Emirates; ^2^ Global Remote Research Scholars Program, St. Paul, MN, United States; ^3^ Punjab Medical College, Faisalabad, Pakistan; ^4^ Bangladesh Medical College, Dhaka, Bangladesh; ^5^ Shifa International Hospital, Islamabad, Pakistan; ^6^ Mental Health Center of Excellence, Al Jalila Children’s Specialty Hospital, Dubai Health, Dubai, United Arab Emirates; ^7^ College of Medicine, Mohammed Bin Rashid University of Medicine and Health Sciences, Dubai Health, Dubai, United Arab Emirates

**Keywords:** eating disorders, adolescents, social media, children, youth, mental health

## Abstract

Eating disorders are on the rise with a significant impact on mental health. Misuse of social media platforms is likely a significant contributing factor. This trend is especially pronounced among adolescents, who are increasingly using social media platforms for communications, building relationships, learning and entertainment. The unsupervised sharing of social media content can have drastic consequences on the physical and psychological wellbeing of youth, which often takes the form of “thinspiration” or “fitspiration”. This probably serves as a trigger for those already struggling with eating disorders. In addition, there is a lack of awareness among youth and adults on ideal knowledge-sharing practices related to an eating disorder. By addressing the unique challenges that social media presents for youth with eating disorders, communities can work towards creating a safer and more supportive online environment on a global scale. At this junction, this article aims to share the current challenges of social media use among adolescents with eating disorders and put forth recommendations for how social media could be used as a tool for positive impact in this population.

## 1 Introduction

Eating disorders (ED) are a group of mental illnesses that can have significant impacts on an individual’s physical and mental health. Adolescents are highly susceptible to developing eating disorders (1). Eating disorders are the third most prevalent chronic illness among adolescents, with only obesity and asthma ranking higher (1). The Diagnostic and Statistical Manual of Mental Disorders, 5th edition (DSM-5) categorizes eating disorders into five groups: Anorexia Nervosa (AN), Bulimia Nervosa (BN), Binge-Eating Disorder (BED), Other Specified Feeding or Eating Disorders (OSFED), and Unspecified Feeding or Eating Disorders (2).

Globally, the prevalence of eating disorders has more than doubled in the past decade, from estimates of 3.5% to 7.8% (3). Forty percent of cases occur among adolescents aged between 15 and 19 (1). The prevalence of disordered eating behavior has been found to be substantial, ranging from 14% to 22% in epidemiological studies (4). AN, BN, and BED have been found to affect 0.3%, 0.9%, and 1.6% of the population, respectively (4). Community samples have also indicated that eating disorders are more prevalent among adolescent females, with 5.7% affected, compared to 1.2% of adolescent males (5).

The onset age of AN, BN, and BED have now shifted to early adolescence, with a median age of 12 years, whereas previous trends indicated mid to late adolescence (1). Furthermore, the biopsychosocial model acknowledges that the increased exposure to social media content that emphasizes on appearance may be linked to greater levels of internalization of beauty standards, thereby leading to increased adoption of such ideals as personal goals for the individual (6). This trend is especially pronounced among adolescents, who are increasingly using social media platforms for communications, building relationships, learning and entertainment.

An estimate of over 90% of adolescents have at least one social media account, and their presence on social media continues to grow (7). In the USA alone, a 2018 study shows that 93% of youth aged 14-22 use social media including platforms like Snapchat, Instagram, Facebook, and Twitter (8). Eighty-one percent of these use it on a daily basis (8). The consensus among most reports is that social media use among adolescents is on the rise (9–11). The growth has been higher since the COVID pandemic that pushed all activities online (9–11). One possible explanation for this potential rise in eating disorders among adolescents could be the influence of celebrity influencers on the body image perception of this age group. This was highlighted in a study where adolescents who had a tendency to develop eating disorders and influenced by popular personalities were found to be significantly associated with body image disturbances (12).

A scoping review from 2021 concludes 4 major factors of influence that eventually lead to disordered eating behaviors (13). These include visual appeal, content dissemination, socialized digital connections and adolescent marketer influencers (13).Visual appeal refers to use of social media by large scale food companies to share images and videos of their food products that promotes eating disordered behaviors. Social media as a platform for content dissemination can also allow for sharing of food recipes and experiences that can promote such behaviors among adolescents with eating disorders.

The impact of socialized digital connections can be a powerful influencer due to the presence of online communities that shape the views and behaviors of individuals through peer pressure and social norms. Adolescent market influencers can further impact on eating behaviors among social media users through their content sharing methods, which can create the perception of influencers being “role models” of food consumption by their viewers. Wilksch, at. el in their study display how increased use of Instagram and Snapchat was associated with significantly higher global Eating Disorder Examination Questionnaire (EDE-Q) scores and disordered eating among girls (14).

Our research question revolves around the relationship between social media use and eating disorders among adolescents. Specifically, we aim to explore how social media platforms contribute to the development and exacerbation of eating disorders in this demographic, as well as the potential for using social media as a positive tool for support and intervention.

Our objectives are threefold. Firstly, we seek to elucidate the challenges posed by social media, such as the promotion of unrealistic beauty standards and the prevalence of content that can trigger or encourage disordered eating behaviors among adolescents. Secondly, we aim to raise awareness about these challenges among both youth and adults, emphasizing the need for informed knowledge-sharing practices related to eating disorders. Lastly, we propose recommendations for change, including the implementation of policies on social media platforms, awareness campaigns, and research efforts to better understand the impact of social media on adolescent mental health.

Our methodology involves a comprehensive review of existing literature, including studies on the prevalence of eating disorders, the impact of social media on body image and disordered eating behaviors, and the potential benefits of awareness campaigns and online communities. Additionally, our recommendations for change are based on a synthesis of findings from the literature and our analysis of current trends in social media use among adolescents.

By addressing these questions, we aim to provide a clear understanding of the scope and methodology of our perspective article, highlighting its significance in addressing the critical issue of eating disorders among adolescents in the digital age.

## 2 Current challenges

Social media platforms often promote unrealistic beauty standards, for instance idealizing lean bodies. These likely push adolescents to engage in unhealthy dieting and excessive exercising (13). Additionally, these contribute to developing self-esteem issues and self-hatred (13).

According to a study based on the use of TikTok (a social media platform) among children and adolescents diagnosed with eating disorders, it was seen that the participants reported participants found anorexia nervosa, termed Pro-Ana and Bulimia nervosa, termed Pro-mia, content frequently without actively searching (15, 16). This may lead the users to imitate behaviors related to ED and non-suicidal self-inflicting injuries (15, 16). This type of content, which often takes the form of “thinspiration” or “fitspiration”, can serve as a trigger for those already struggling with eating disorders. “Thinspiration” and “fitspiration” are terms that refer to content, often disseminated through social media, intended to inspire individuals toward achieving thinness or fitness, respectively. In the context of eating disorders, these concepts can contribute to unhealthy body image ideals. Thinspiration typically focuses on promoting extreme thinness as an aesthetic goal, while fitspiration emphasizes achieving a fit and muscular physique (17). It can even encourage the development of these tendencies in vulnerable children. Furthermore, in a study done on adolescents in a school, it was seen that children with disordered eating were more likely to get bullied by their peers (18). This in turn may have a drastic effect on their mental health (18).

In addition to this, there is a lack of awareness among youth and adults on ideal knowledge-sharing practices related to an eating disorder. In a cross-sectional study done among families with children with eating disorders, it was seen that more than half of the parents did not have any knowledge regarding the eating disorder recovery websites (19). A significant percentage of teenagers frequently visited the pro-eating disorders website and 96% of them reported learning new weight loss or purging technique (19).

In the past few years a trend of appreciating all body types has been started on social media platforms (20). This is referred to as body-positive content and it encourages showing unedited, unenhanced, and unfiltered photos. Although this trend has a positive intention, some studies have shown that such exposures may be linked to a higher level of self-objectification (21). For instance, body-positive content emphasized on accepting various body appearances and shapes that are unique to each individual. However, as the focus is still on the body, self-objectification is expected to increase especially in women. In addition, cultural pressures as seen in many Western societies have contributed to women viewing themselves as “objects”. This eventually has a negative impact on physical and emotional wellbeing (22). Furthermore, social media has been observed as a communication medium for negative self-harm behaviors, which in turn creates a cycle of amplifying or encouraging such behaviors through shared videos (23).

## 3 Discussion

### 3.1 Recommendations for change

#### 3.1.1 Regulations on social media-use among adolescents

It is imperative that social media platforms implement policies that acknowledge the negative impact of social media on body image and resultant disordered eating behaviors among adolescents. Such policies should include measures to protect users’ personal data and encourage safe reporting of negative content online. A dedicated mental health taskforce, including mental health professionals, should be put in place to oversee the implementation of these policies and monitor their effectiveness in creating a positive online environment. Such a task force would ensure that the policies are adequately enforced and would provide insights into how social media platforms can improve their policies to better protect the mental health of adolescents with eating disorders.

An example of such initiative is seen by the announcement of an action plan by the United States Government, which is focused on protecting youth mental health, safety, and privacy online by collaborating with departments of health and human services, commerce, education, and justice. This enables an integrated approach to monitoring potentially harmful social media activities as well as creating tailored education initiatives to promote healthy social media usage among adolescents (24). By implementing such measures, social media platforms can play a vital role in promoting positive mental health among adolescents with eating disorders and creating a safe online environment for all users. At a personal level, the role of parents or caregivers in regulation of social media use in this population should not be underestimated. There is a need for increased discussion and engagement between parents and adolescents with eating disorders to prevent and manage the potential risks of social media use for their wellbeing.

#### 3.1.2 Awareness campaigns and online communities

It is important for experts in the mental health field and official health organizations to spearhead awareness campaigns. These should aim at educating adolescents about eating disorders and promoting healthy conversations about these topics online. Studies have shown the impact of use of social media on eating disorders and body image issues (25). These campaigns can help reduce the stigma surrounding eating disorders and promote early detection and treatment. A review of eating disorders related hashtag campaigns during the Eating Disorders Awareness Week on the X platform (formerly known as Twitter) concluded that awareness campaigns effectively promoted posting about eating disorders (26). To further promote awareness, education about eating disorders should be integrated into social media platforms as well as school curricula, providing students with a solid foundation of knowledge and resources to help identify and address these issues (26). Anti-bullying efforts should also be incentivized among youth online to ensure that social media platforms remain a safe and supportive space for all users. This can be done by adding virtual badges for online profiles that are actively engaged in promoting anti-bullying content on social media.

Additionally, workshops and training exercises can be arranged to train mental health influencers on social media advocacy for eating disorders. These exercises can aid influencers understand the importance of promoting positive body image and self-love. Additionally, online educational forums can also create a space for youth to discuss eating disorders and their impact on mental health, thereby promoting a supportive online community that values mental health and wellness. By working together, experts, educators, influencers, and adolescents can help create a safer, more supportive online environment for all.

#### 3.1.3 Research and funding

Literature shows the need for research and funding aimed towards understanding the impact of social media on overall mental health and eating disorders among youth. This can help in developing effective interventions and guidelines for social media use. Such research can help identify the specific features of social media that contribute to disordered eating behaviors, including the role of algorithms, body shaming, and the prevalence of idealized body images.

Furthermore, studying the impact of social media algorithms on disordered eating behaviors is crucial. Algorithms can influence what content users are exposed to, which can further reinforce harmful beauty standards and promote disordered eating behaviors. By understanding the role of algorithms, researchers can develop interventions that aim to address the negative impact of these algorithms and promote healthy social media use among youth.

## 4 Conclusion

Eating disorders are on the rise with a significant impact on mental health. Misuse of social media platforms is likely a significant contributing factor. There is also a great potential in utilizing these platforms as positive tools for youth struggling with eating disorders. Taking this double-edged sword into consideration, investing in research and funding for understanding the impact of social media on eating disorders can help develop early interventions and guidelines that promote healthy social media use among youth. By addressing the unique challenges that social media presents for youth with eating disorders, communities can work towards creating a safer and more supportive online environment on a global scale.

## Data availability statement

The original contributions presented in the study are included in the article/supplementary material. Further inquiries can be directed to the corresponding author.

## Author contributions

FN: Writing – original draft, Writing – review & editing. MR: Writing – original draft. NB: Writing – original draft. AS: Writing – original draft, Writing – review & editing. ZA: Writing – review & editing. HD: Writing – review & editing. MS: Writing – original draft, Writing – review & editing.
